# Oxytocin Protects against Stress-Induced Cell Death in Murine Pancreatic β-Cells

**DOI:** 10.1038/srep25185

**Published:** 2016-05-04

**Authors:** Sayaka Watanabe, Fan-Yan Wei, Tomomi Matsunaga, Nanami Matsunaga, Taku Kaitsuka, Kazuhito Tomizawa

**Affiliations:** 1 Department of Molecular Physiology, Faculty of Life Sciences, Kumamoto University, Kumamoto 860-8556, Japan

## Abstract

Oxytocin (Oxt) is a key neuropeptide that regulates maternal behaviors as well as social behaviors in mammals. Interestingly, recent studies have shown that the impairment of Oxt signaling is associated with the disturbance of metabolic homeostasis, resulting in obesity and diabetes. However, the molecular mechanism by which Oxt signaling controls metabolic responses is largely unknown. Here, we report that Oxt signaling attenuates the death of pancreatic beta cells in islets exposed to cytotoxic stresses. The protective effect of Oxt was diminished in islets isolated from oxytocin receptor knockout (Oxtr^−/−^) mice. Oxtr^−/−^ mice developed normally, but exhibited impaired insulin secretion and showed glucose intolerance under a high-fat diet. Mechanistically, the deficiency of Oxtr impaired MAPK/ERK-CREB signaling, which exaggerated the endoplasmic reticulum stress response and ultimately increased the death of beta cells in pancreatic islets under stressed conditions. These results reveal that Oxt protects pancreatic beta cells against death caused by metabolic stress, and Oxt signaling may be a potential therapeutic target.

Oxytocin (Oxt) is a multifunctional hormone consisting of a mature polypeptide of nine amino acids^1^. In mammals, Oxt is produced in subpopulations of neurons in the supraoptic nuclei (SON) and paraventricular nuclei (PVN) of the hypothalamus^1^. The hormone is transported to the nerve terminals by axonal transport and released from the posterior pituitary gland^1^. The central physiological functions of oxytocin are to regulate maternal^2^, emotional^3^, affiliative^4^, and sexual^5^ behaviors, as well as spatial and social cognition^6^,^7^. Oxt binds to G_q/11_α proteins coupled to the oxytocin receptor (Oxtr) predominantly expressed in uterine smooth muscle and myoepithelial cells^8^. The activation of Oxtr induces an increase in calcium from both intracellular calcium stores and extracellular calcium influx^9^. The increase in calcium triggers the activation of calcium-dependent protein kinases, which finally induces smooth muscle contraction and leads to parturition and lactation^8^.

Besides the above physiological functions in mammals, Oxt has recently been emerging as a key component of metabolic homeostasis. Treatment with Oxt was reported to stimulate glucose oxidation and lipogenesis in adipocytes^10^. In the pancreas, a physiological level of Oxt is detected in both humans and rodents^11^. The treatment of isolated pancreatic islets with Oxt stimulates the release of glucagon as well as insulin^12^. The involvement of Oxt signaling in metabolism has been confirmed in a mouse model with the genetic deletion of either *Oxy* or *Oxtr*. Both *Oxt*-null and *Oxtr*-null mice had normal food intake patterns but developed late-onset obesity^13^,^14^. The involvement of Oxt signaling in metabolic homeostasis has also been supported by human studies. CD38, a membrane ADP-ribosyl cyclase, regulates Oxt secretion. Interestingly, single nucleotide polymorphisms of the CD38 gene have been implicated in the development of diabetes^15^. This observation has been validated in *CD38*-null mice with an ICR genetic background^16^,^17^. *CD38*-null mice exhibited impaired insulin secretion and an elevated plasma glucose level. Furthermore, recent epidemic studies showed that breast-feeding is likely to be associated with a lower incidence of type 2 diabetes^18^,^19^. Taken together, these results suggest that Oxt signaling has a beneficial effect in metabolic homeostasis.

Given the important role of Oxt signaling in metabolism, a large number of studies have aimed to understand the potential functions of Oxt in adipocytes as well as in acute insulin secretion^10^,^11^. However, the molecular mechanism by which Oxt signaling regulates the cellular homeostasis of pancreatic islets has been insufficiently explored. In the present study, we investigated the anti-cell death effect of Oxt signaling *in vitro* and *in vivo* by cell biological and genetic approaches.

## Results

### Inhibitory effect of Oxt on stress-induced cell death of pancreatic islets

**Figure 1:**
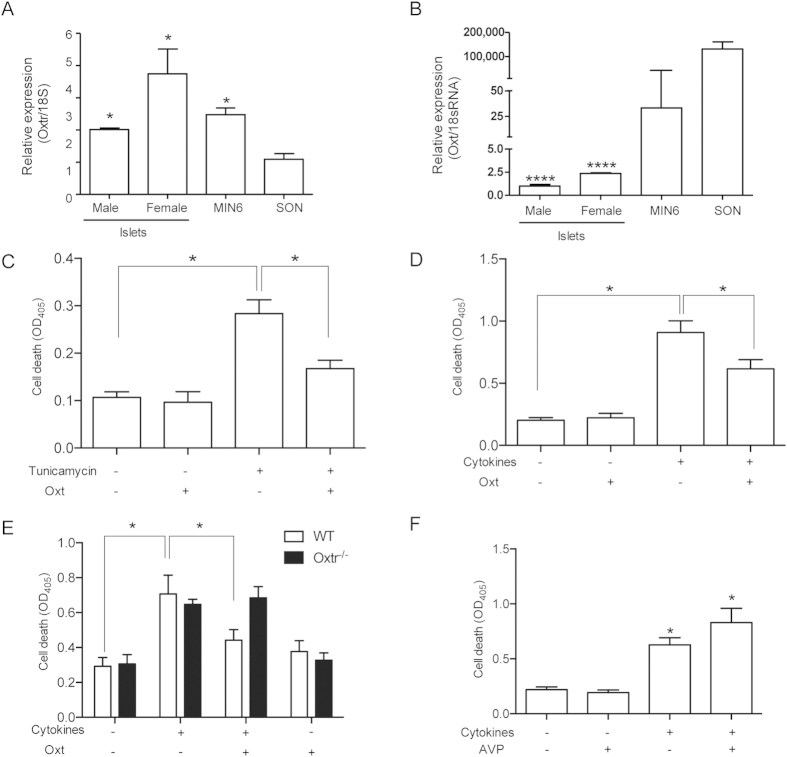
Oxt attenuates cell death in pancreatic islets. (**A,B**) Relative expressions of *Oxt* (**A**) and *Oxtr* (**B**) mRNAs in the pancreatic islets of male and female wild-type mice; MIN6 cells and SON of male wild-type mice are shown. *P < 0.05 and ****P < 0.0001 versus SON by one-way ANOVA. n = 5–8. (**C,D**). Cell death was measured in islets isolated from male mice treated with 2 ng/mL tunicamycin (**C**) or a cytokine mixture (**D**) in the presence of 100 pM Oxt for 24 hours. *P < 0.05, n = 8 for (**C**), *P < 0.05, n = 12 replicates for (**D**) by Student’s *t*-test adjusted for multiple comparison. (**E**) Cell death was measured in islets isolated from wild-type (WT) and Oxtr^−/−^ mice treated with 100 pM oxytocin and a cytokine mixture for 24 hours. *P < 0.05 by Student’s *t*-test. n = 8. (**F**) Cell death was measured in islets isolated from wild-type mice treated with 1 nM vasopressin and a cytokine mixture for 24 hours. n = 12.

To investigate the role of Oxt signaling in pancreatic islets, we first examined the expression levels of *Oxt* and *Oxtr*. There was a large amount of *Oxtr* mRNA in the pancreatic islets isolated from both male and female mice as well as in the murine pancreatic beta cell-derived cell line MIN6 (Fig. 1A). The expression level in the pancreatic islets and MIN6 cells was higher than that in mouse SON. In contrast, only a trace of *Oxt* mRNA was detected in the islets and MIN6 cells compared with mouse SON (Fig. 1B), suggesting that Oxt does not act through a paracrine pathway in pancreatic islets.

Given the impaired metabolic homeostasis in mice and humans with deficiency of Oxt signaling^13^,^14^,^15^, we hypothesized that Oxt signaling is involved in anti-cell death signaling in pancreatic islets. To investigate this hypothesis, pancreatic islets were isolated from male mice and then treated with Oxt in the presence of various metabolic stressors including tunicamycin (Fig. 1C), cytokines (Fig. 1D), and palmitate (Fig. 1E). These cytotoxic reagents significantly induced cell death in isolated pancreatic islets (Fig. 1C–E). Treatment with 100 pM Oxt, a physiological concentration in plasma, effectively attenuated the stressor-induced cell death (Fig. 1C–E). To further elucidate the inhibitory effect of Oxt, we isolated pancreatic islets from mice with deficiency of Oxtr (Oxtr^−/−^) and treated them with cytokines. As expected, the protective effect of Oxt against cytokine-induced cell death was no longer observed in the islets isolated from Oxtr^−/−^ mice (Fig. 1F). Oxtr is selective for Oxt in general, but is also capable of binding to vasopressin (Avp) with a low affinity^20^. To verify the specificity of Oxt signaling, pancreatic islets were treated with 1 nM Avp and cytokines. However, treatment with Avp failed to prevent cytokine-induced cell death (Fig. 1G).

### Oxtr^−/−^ mice exhibit normal metabolic homeostasis

**Figure 2:**
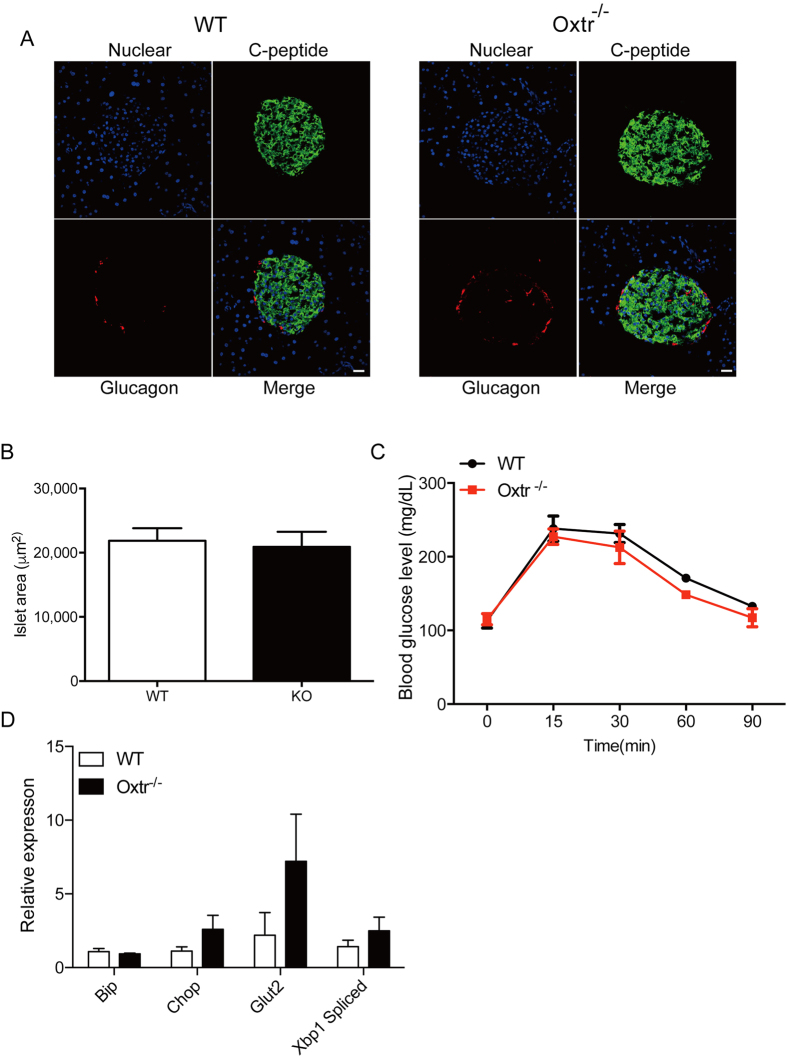
Normal glucose metabolism in Oxt^−/−^ mice. (**A**) Morphological examination of pancreatic islets of WT and Oxtr^−/−^ mice. Pancreatic islets were stained with anti-glucagon and anti-C-peptide antibodies. Nuclei were stained with DAPI. Scale bars = 50 μm. (**B**) The islet area of Oxtr^−/−^ mice did not differ from that of WT mice. n = 22. (**C**) Glucose tolerance tests were performed in WT and Oxtr^−/−^ mice. n = 3–5. (**D**) The expression levels of ER stress-related genes were examined by quantitative PCR. Note that no significant difference was observed between WT and Oxtr^−/−^ mice. n = 5.

Given the protective role of Oxt signaling, deficiency of Oxtr might impair cellular homeostasis as well as insulin-mediated glucose metabolism in pancreatic islets, which will collectively lead to late-onset obesity. However, there was no morphological change in pancreatic islets of Oxtr^−/−^ mice when compared with the wild-type control littermates (Fig. 2A,B). There was no clear decrease of insulin-positive or glucagon-positive cells in Oxtr^−/−^ islets (Fig. 2A). Accordingly, the glucose levels of Oxtr^−/−^ mice were comparable with those of WT mice during the glucose tolerance test (Fig. 2C). Furthermore, the food intake and body weight of Oxtr^−/−^ mice were also the same as those of WT mice (data not shown). In agreement with these observations, the expression levels of endoplasmic reticulum stress-related genes, which are involved in cell death signaling, were comparable between WT and Oxtr^−/−^ mice (Fig. 2D). Consistent with a previous study^13^, these results suggest that a deficiency of Oxt signaling does not immediately lead to pathological phenotypes.

### Oxtr^−/−^ mice develop glucose intolerance under high-fat diet

**Figure 3:**
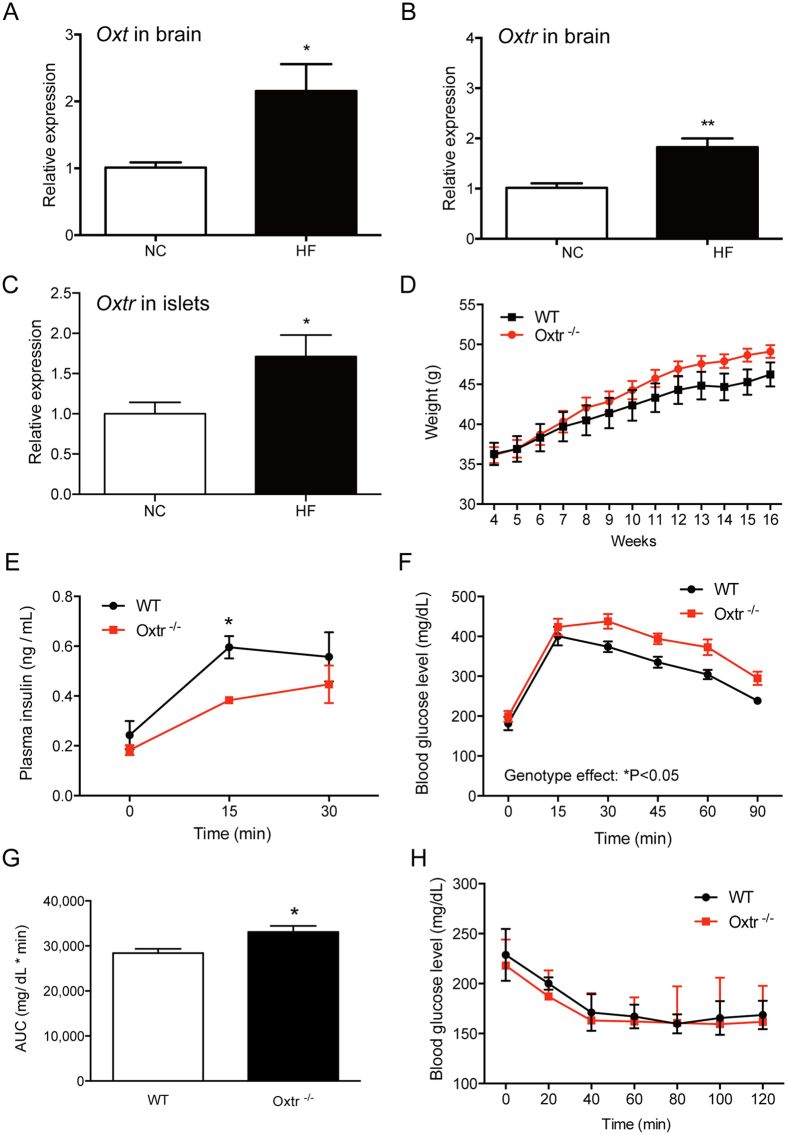
High-fat diet impairs glucose metabolism in Oxtr^−/−^ mice. (**A,B**) WT mice were fed with a high-fat diet for 19 weeks. The expression levels of *Oxt* (**A**) and *Oxtr* (**B**) in the brain were examined by quantitative PCR. n = 5~6. *P < 0.05, **P < 0.01 by Student’s *t*-test. (**C**) The expression level of *Oxtr* in pancreatic islets of WT mice fed a high-fat diet was examined. n = 5~8. *P < 0.05 by Student’s *t*-test. (**D**) WT and Oxtr^−/−^ mice at 12 weeks old were fed a high-fat diet. The change in body weight is shown. n = 13. (**E**) Mice fed a high-fat diet for 18 weeks were challenged with glucose, and the plasma insulin levels at indicted time-points were measured. *P < 0.05 by repeated measures two-way ANOVA. n = 4–5. (**F**) Glucose tolerance tests were performed in mice fed a high-fat diet for 18 weeks. *P < 0.05 by repeated measures two-way ANOVA. n = 4–5. (**G**) The area under the curve (AUC) corresponding to the glucose tolerance test (**F**) was calculated. *P < 0.05 by Student’s *t*-test. n = 4–5. (**H**) The insulin tolerance test was performed in mice fed a high-fat diet for 18 weeks. n = 10.

Oxt has been implicated in metabolic regulation in peripheral tissues such as adipocytes and the pancreas^10^,^11^,^12^. Interestingly, when wild-type mice were fed a high-fed diet, there was a significant increase in both *Oxt* and *Oxtr* levels in the brain, and the *Oxtr* level in the islets (Fig. 3A–C). However, the circulating blood Oxt level did not differ between WT and Oxtr^−/−^ mice (data not shown). The increase of *Oxt* and *Oxtr* in both central and peripheral tissues suggests that Oxt signaling is actively involved in the adaptive response to metabolic stress. This perspective prompted us to challenge Oxtr^−/−^ mice with metabolic stress such as a high-fat diet. The body weight of Oxtr^−/−^ mice was slightly heavier than that of WT mice after high-fat diet feeding for 16 weeks (Fig. 3D). To investigate glucose metabolism, Oxtr^−/−^ mice fed the high-fat diet were subjected to the glucose tolerance test. Insulin secretion after glucose challenge was significantly impaired in Oxtr^−/−^ mice (Fig. 3E). Accordingly, the reduction of the plasma glucose level was significantly attenuated in Oxtr^−/−^ mice when compared with that in WT mice (Fig. 3F–G). To examine the potential impact of metabolic stress on insulin sensitivity, the insulin tolerance test was performed in WT and Oxtr^−/−^ mice. However, insulin sensitivity in Oxtr^−/−^ mice fed the high-fat diet was comparable with that in WT mice (Fig. 3H). These results suggest that insulin secretion from pancreatic islets was selectively impaired in Oxtr^−/−^ mice with chronic metabolic stress.

### Stress-induced apoptosis in Oxtr^−/−^ pancreatic islets

**Figure 4:**
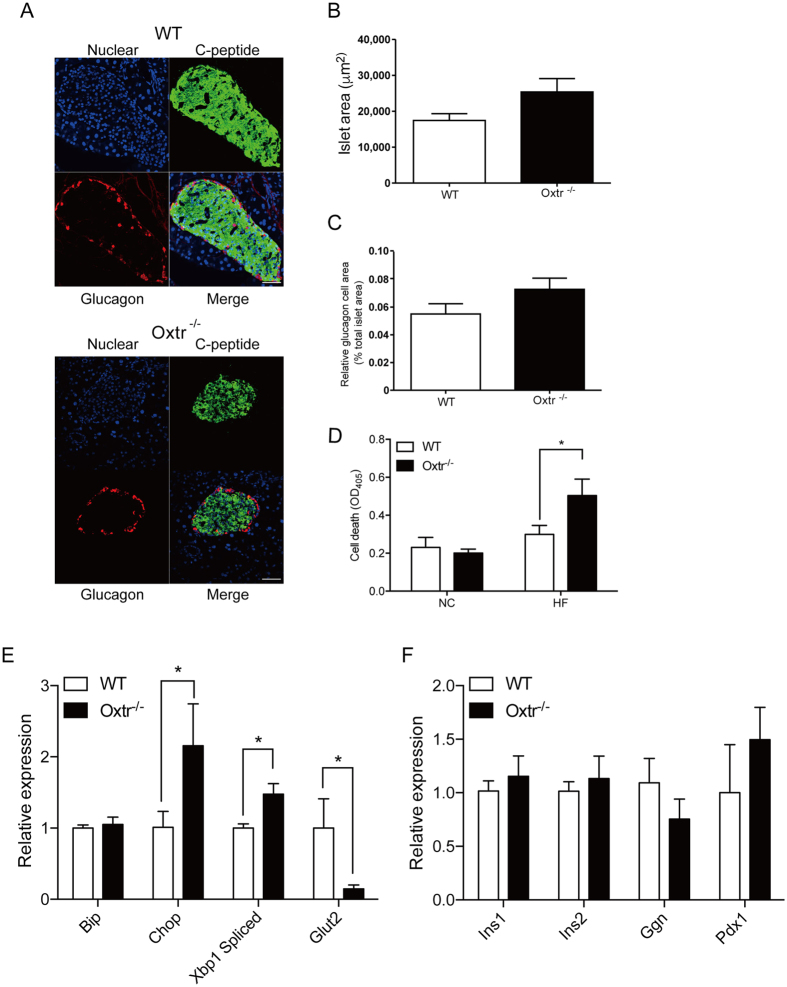
Oxtr^−/−^ mice exhibit enhanced ER stress response under metabolic stress. (**A**) Morphological examination of pancreatic islets in WT and Oxtr^−/−^ mice fed a high-fat diet for 16 weeks. Pancreatic islets were stained with anti-glucagon (red) and anti-C-peptide (green) antibodies. Nuclei were stained with DAPI (blue). Scale bars = 50 μm. (**B**) The islet area of WT and Oxtr^−/−^ mice fed a high-fat diet for 16 weeks was examined. n = 21. (**C**) The relative glucagon cell area was calculated by dividing the glucagon cell area by the total islet area. n = 21. (**D**) Cell death in the islets of WT and Oxtr^−/−^ mice fed normal chow (NC) or a high-fat diet (HF) for 16 weeks. *P < 0.05 by Student’s *t*-test. n = 5. (**E,F**) Expression levels of genes related to the ER stress response (**F**) and islet functions (**G**) were examined. *P < 0.05 by Student’s *t*-test. n = 5 each.

Metabolic stress is one of the major triggers of chronic inflammation that causes cell death^21^. Considering the anti-cell death role of Oxt signaling in pancreatic islets, we suspected that the high-fat diet-induced glucose intolerance in Oxtr^−/−^ mice might be due to the augmentation of cell death in Oxtr^−/−^ islets. Morphological examination of pancreatic islets revealed no difference between WT and Oxtr^−/−^ mice (Fig. 4A). Neither the size of islets nor the number of glucagon cells significantly differed between WT and Oxtr^−/−^ mice (Fig. 4B,C). To examine cell death in stressed mice, we isolated pancreatic islets from the stressed mice and examined cell death. As expected, there was a significant increase of cell death in Oxtr^−/−^ islets (Fig. 4D). Accordingly, TUNEL-positive cells were observed in Oxtr^−/−^ islets (Fig. 4E).

Next, we investigated the molecular mechanism underlying stress-induced cell death in Oxtr^−/−^ islets. Metabolic stress is a strong inducer of endoplasmic reticulum stress (ER stress), which leads to cell death by the upregulation of proapoptotic genes such as *Chop* and *Xbp1*^21^. We thus investigated the expression levels of ER stress-related genes such as *Bip*, *Chop*, *Xbp1,* and *Glut2*. Indeed, there was a significant increase of *Chop* and *Xbp1* levels in stressed Oxtr^−/−^ islets (Fig. 4F). Furthermore, there was a significant decrease of *Glut2* levels, reflecting the impaired glucose response in Oxtr^−/−^ islets (Fig. 4F). On the other hand, the expression levels of genes related to pancreatic islet functions, such as *Ins1/2*, *Pdx1,* and *Glucagon,* remained unchanged between WT and Oxtr^−/−^ mice (Fig. 4G).

**Figure 5:**
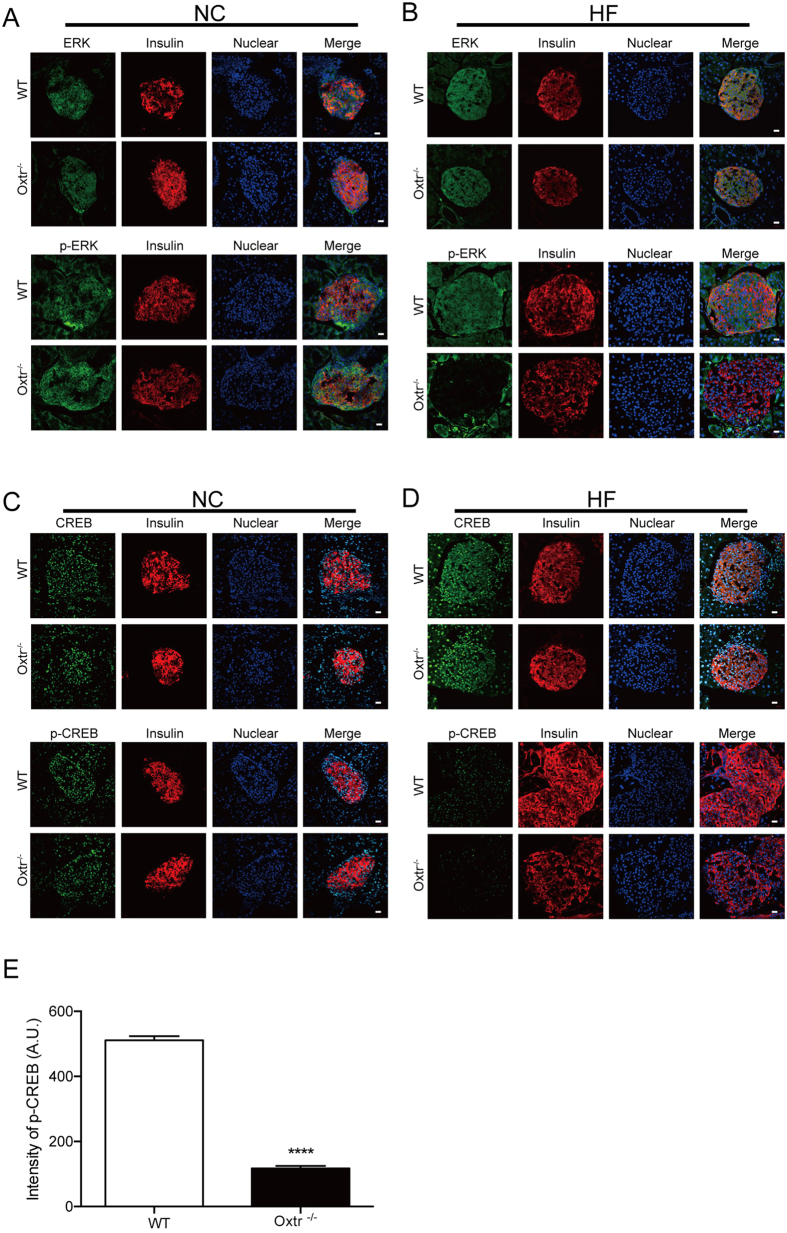
Impaired ERK-CREB signaling in Oxtr^−/−^ mice. (**A–D**) Pancreatic sections of mice fed normal chow (NC) (**A**) or a high-fat diet (HF) (**B**) were stained with anti-insulin antibody in combination with anti-ERK anti-phospho-ERK (P-ERK). Note that the p-ERK signal was decreased in Oxtr^−/−^ mice fed HF. (**C,D**) Pancreatic sections of mice fed NC (**C**) or HF (**D**) were stained with anti-CREB or anti-phospho-CREB (P-CREB) antibodies. Note that the p-CREB signal was decreased in Oxtr^−/−^ mice fed HF. Nuclei were stained with DAPI. Scale bars = 20 μm. (**E**) The p-CREB levels in islets of WT and Oxtr^−/−^ mice fed a high-fat diet (**D**) were quantified by measuring the intensity of p-CREB in individual nuclei. n = 283 cells for WT, n = 147 cells for KO. ****P < 0.0001 by Student’s *t*-test.

Finally, we investigated the molecular pathway that contributes to the stressed-induced cell death in Oxtr^−/−^ pancreatic islets. The binding of Oxt to Oxtr initiates a variety of downstream signaling pathways, including the mitogen-activated protein kinase (MAPK)/extracellular signal-regulated kinase (ERK)-cAMP response element binding protein (CREB) pathway, which has recently been implicated in the ER stress response^22^. To investigate the potential involvement of ERK-CREB signaling in pancreatic islets, the active phosphorylated forms of ERK1/2 and CREB were immunostained in pancreatic islets of WT and Oxtr^−/−^ mice fed with normal chow or a high-fat diet (Fig. 5A–D). There was a marked reduction of phosphor-ERK1/2 and phosphor-CREB in Oxtr^−/^ mice fed a high-fat diet (Fig. 5B,D). These results thus suggest that the ERK-CREB signaling was impaired in Oxtr^−/−^ mice under chronic metabolic stress.

## Discussion

The present study revealed a beneficial role of Oxt signaling in response to cytotoxic stimulation in pancreatic islets. The activation of Oxt signaling attenuated cell death induced by cytotoxic cytokines in pancreatic islets. The anti-cell death effect of Oxt was further demonstrated in mice with a deficiency of Oxtr. The Oxtr^−/−^ mice fed the high-fat diet exhibited increased apoptosis in pancreatic islets, which results in impaired insulin secretion as well as glucose intolerance. Consistent with our study, the protective role of Oxt signaling in peripheral tissue has emerged from recent studies^23^,^24^,^25^. The application of Oxt protected cardiomyocytes from apoptosis in the rat heart subjected to temporary ischemia as well as in obese diabetic db/db mice^23^,^24^,^25^. These results thus suggest that Oxt signaling is actively involved in protection of the cellular homeostasis of pancreatic islets, which ultimately contributes to the metabolic balance.

Despite the accumulating evidence showing the beneficial role of Oxt signaling, the molecular mechanism of the anti-cell death effect of Oxt has been largely unexplored. Oxy binds to Oxtr coupled with Gq, which leads to calcium mobilization^1^. In neurons, the Oxt-evoked calcium signaling transduces to the MAPK/ERK cascade, which further leads to the activation of CREB and consequently contributes to learning and memory^7^. The ERK-CREB cascade promotes cell survival by both the ERK-dependent phosphorylation of prosurvival proteins and CREB-dependent transcription of prosurvival genes^22^. Given the marked decrease of phosphorylation of ERK and CREB in the Oxtr^−/−^ islets, it is conceivable that the loss of the Oxt-ERK-CREB cascade contributes to the stress-induced apoptosis.

A unique finding in this study was the involvement of Oxt signaling in the ER stress response in pancreatic islets. The levels of canonical ER stress genes, such as *Xbp1* and *Chop*, were significantly upregulated in the stressed Oxtr^−/−^ islets, suggesting that Oxt signaling suppresses the ER stress response. However, the molecular mechanism by which Oxt signaling is associated with the ER stress response remains unknown. Recently, some pieces of evidence have shown that CREB is involved in the ER stress response through competitive association with activating transcription factor 6 alpha (ATF6α), which is an integrator of ER stress^26^. Both CREB and ATF6a bind to a common transcription factor named CREB-regulated transcription coactivator 2 (CRTC2) in a mutually exclusive manner. A decrease of CREB would result in a reciprocal increase of the ATF6-CRTC2 complex, which promotes the expression of ER stress response genes including *Xbp1* and *Chop*^26^. It is likely that the reduction of phospho-CREB in Oxtr^−/−^ islets reciprocally enhances the ATF6-CRTC2-mediated ER stress response, which consequently promotes apoptosis.

Given the anti-cell death effect of Oxt signaling, it is worthwhile to mention the potential role of Oxt signaling in Alzheimer’s disease (AD). AD is a neurodegenerative disease characterized by unique histopathological and biological abnormalities, including cell death, amyloid-β deposits, and memory loss^27^. A growing body of evidence links type 2 diabetes to the development of AD^28^. Interestingly, the nucleus basalis of Meynert in the forebrain, which contains a number of cholinergic neurons that are degenerated in AD patients, exhibits a high expression level of OXTR in humans^29^. It is likely that the dysregulation of Oxt signaling is involved in the development of AD^30^.

Given the beneficial effect of Oxt in metabolism, it has been applied to animal models of diabetes^31^. Oxt administration effectively reduced obesity and obesity-related glucose metabolism in diabetic rodents^32^,^33^. Moreover, a clinical trial using Oxt as an anti-obesity drug was recently conducted^34^. The Oxt therapy showed some positive effects on weight control^34^. In the present study, we observed impaired glucose tolerance, but the insulin sensitivity was not significantly affected in Oxtr^−/−^ mice. These results suggest that Oxt signaling mainly acts on the pancreatic islets to maintain the hormonal balance. Furthermore, the protective effect of Oxt signaling on islets might explain the positive metabolic outcomes in the previous Oxt therapies. Further study using human pancreatic islets will be needed to elucidate the potential role of Oxt signaling in humans.

Taken together, our results reveal that Oxt signaling is critical for the cell survival of pancreatic islets under inflammatory metabolic stress conditions. These findings thus reveal the mechanistic basis for the potential of Oxt therapy to treat diabetes.

## Methods

### Animals

The *Oxtr*-deficient mice (T583) described previously^35^ were used in all experiments. All mouse strains were backcrossed to achieve a C57BL/6 genetic background for more than 6 generations. Unless specified otherwise, all mice used in this study were male. Oxtr knockout (Oxtr^−/−^) mice were generated by obtaining heterozygous mice crossed with each other, and the littermates of the wild-type were used as a control. Animals were housed at 25˚C with 12-hour light/12-hour dark cycles. High-fat chow (D12451, 45% kcal% fat) was purchased from Research Diets (New Brunswick, NJ, USA). Normal chow (CE-2, 4.6% kcal% fat) was purchased from CLEA Japan (Tokyo, Japan). All animal procedures were approved by the Animal Ethics Committee of Kumamoto University (approval ID: A27-037). All procedures were carried out in accordance with the approved guidelines.

### Gene expression analysis

Islets were isolated from Oxtr^−/−^ and wild-type mice by intraductal collagenase (Liberase TL grade; Roche) digestion followed by hand-picking, as described previously^36^. The whole brains were rapidly excised from mice and sliced at a thickness of 500 μm using VT1200S (Leica). The region corresponding to the supraoptic nuclei was then punched out and subjected to RNA purification. Total RNA of islets and brain tissues were purified using an RNeasy Mini Kit (Qiagen) according to the manufacturer’s protocol. A PrimerScript RT Reagent Kit (TAKARA) was used to generate cDNA. Quantitative real-time PCRs were performed using either a TaqMan Gene Expression Kit (Oxt; Mm00726655-s1, Oxtr; Mm01182684-m1 from Applied Biosystems) or SYBR Pre-mix Ex Taq kit (Applied Biosystems). Primers for detection of the ER stress response and islet function were described previously^36^. 18 S rRNA was used as a reference gene for normalization.

### Cell death assay

Islets isolated from wild-type and Oxtr^−/−^ mice were isolated and cultured in RPMI medium supplemented with 5% FBS overnight. Subsequently, only size-matched and well-shaped islets were transferred to a 12-well plate (20 islets/well), with each well represents one replicate. The islets were treated with 100 pM OXT (Sigma), 1 nM AVP (Sigma), 2 ng/mL Tunicamycin (Sigmga-Aldrich), or a cytokine mixture [IL1-β (50 U/mL) •TNF-α (1 × 10^3^ U/mL) •INF-α (1 × 10^3^ U/mL), Wako] for 24 hours. Cell death was measured by the Cell Death Detection ELISA Assay Kit (Roche) according to the manufacturer’s protocol. The cell death level was measured as the absorbance at 405 nm with respect to a substrate solution blank. The experiment was repeated for three times. The n number represents the total number of replicates from these independent experiments.

### Measurement of blood glucose and insulin levels

For the glucose tolerance test, mice at 15~16 weeks of age were fasted for 14 hours (8:00 pm to 10:00 am) or 7 hours (9:00 am to 4:00 pm), followed by the intraperitoneal injection of glucose at a dose of 1 g/kg body weight. For the insulin tolerance test, mice were fasted for 14 hours and injected with human insulin (Humulin R, Eli Lilly) at a dose of 1 unit/kg body weight. Blood glucose was determined at the indicated time-points by a glucometer (ACCU-CHEK, Aviva; Roche). Plasma insulin levels were determined using the Mouse Insulin ELISA kit (Shibayagi) according to the manufacturer’s instructions.

### Immunohistochemical analysis

For immunohistochemical examination, mice at 15~16 weeks of age were perfused with 4% paraformaldehyde and subjected to sectioning using Cyrostat (Leica). Pancreatic sections were stained using anti-Insulin (HyTest), anti-C peptide (Cell Signaling), anti-ERK, anti-Phospho-ERK (Cell Signaling), anti-CREB anti-Phospho-CREB (Cell Signaling), and anti-glucagon (Sigma-Aldrich) antibodies. All antibodies were used at 1:500 dilutions. DAPI solution (Dojindo) was used to stain nuclei. Images were obtained using a FV1000 confocal microscope (Olympus). For measurement of the islet area, randomized pancreatic sections were obtained from 3 mice, and stained with anti-insulin and anti-glucagon antibodies. The insulin-positive and glucagon-positive areas were measured as islet areas using Image J software (NIH), as described previously^37^.

### Statistical analysis

All data are presented as the mean ± SEM. Statistical analyses were performed using Prism 6 Software (GraphPad Software). The unpaired Student’s *t*-test was used to test the differences between two groups. Analysis of variance (one-way ANOVA, two-way ANOVA, and repeated measures two-way ANOVA) was used to test the difference among multiple groups followed by a *post-hoc* examination of the *P*-value between two groups. A 2-tailed *P*-value of 0.05 was considered significant.

## Additional Information

**How to cite this article**: Watanabe, S. *et al.* Oxytocin Protects against Stress-Induced Cell Death in Murine Pancreatic β-Cells. *Sci. Rep.* **6**, 25185; doi: 10.1038/srep25185 (2016).
